# Effect of CeO_2_ on Microstructure and Synthesis Mechanism of Al-Ti-C Alloy

**DOI:** 10.3390/ma11122508

**Published:** 2018-12-10

**Authors:** Wanwu Ding, Taili Chen, Xiaoyan Zhao, Chen Xu, Xingchang Tang, Jisen Qiao

**Affiliations:** 1 State Key Laboratory of Advanced Processing and Recycling of Nonferrous Metals, Lanzhou 730050, China; chentaili411@163.com (T.C.); zhaoxy411@163.com (X.Z.); xuchencl@163.com (C.X.); tangxingchanglut@163.com (X.T.); jsqiaocl@163.com (J.Q.); 2 School of Materials Science and Engineering, Lanzhou University of Technology, Lanzhou 730050, China

**Keywords:** CeO_2_, Al-Ti-C alloy, quenching experiment, microstructure characteristics, synthesis mechanism

## Abstract

The effects of CeO_2_ on the microstructure and synthesis mechanism of Al-Ti-C alloy were investigated by quenching experiment method, while using Al powder, Ti powder, graphite powder, and CeO_2_ powder as main raw materials. The results showed that the addition of CeO_2_ was favorable for promoting the formation of TiC particles in Al-Ti-C systems. With CeO_2_ contents increasing, the distribution of TiC particles were more homogeneous, and the rare earth phase Ti_2_Al_20_Ce was formed. CeO_2_ had little effect on the synthesis of Al_3_Ti particles in Al-Ti-C systems, but had a significant effect on the synthesis of TiC particles. In the Al-Ti-C system, TiC is mainly formed by the reaction of dissolved [Ti] and solid C in the melt. While in the Al-Ti-C-Ce system, CeO_2_ reacts with C and O_2_ to form CeC_2_ firstly, and then CeC_2_ reacts with dissolved [Ti] to form TiC. Based on thermodynamic calculation and microstructure analysis in the process of reaction, a macroscopic kinetic model of Al-Ti-C-Ce system reactions was proposed in this paper.

## 1. Introduction

Grain refinement of aluminum and its alloys can significantly improve the mechanical properties, casting properties, deformation processing properties, and surface quality of the materials [1,2]. As compared to Al-Ti-B refiner, Al-Ti-C refiner is considered as the most promising grain refiners for aluminum and its alloy due to the small size of TiC particles, low aggregation tendency in aluminum melt, and difficulty in poisoning with element such as Zr and Cr [3,4,5,6,7]. Unfortunately, the key issue in the production of Al-Ti-C alloy is that the wet-ability between carbon and Al melt is poor, which makes the formation of TiC difficult [8,9,10]. It has been reported that the rare earth metals have certain refining ability and catalytic effect [11,12], which play an important role in promoting the synthesis of fine TiC particles in the preparation of Al-Ti-C alloy, and thus, Al-Ti-C-RE alloys with better refining efficiency than Al-Ti-C have been developed [13,14,15,16]. Although such alloys have been extensively studied, the refining properties of the prepared Al-Ti-C-RE alloys are not sufficiently stable [17,18]. So far, it has not been widely used in the aluminum and its alloy refining industry.

The main influence on the efficiency of Al-Ti-C grain refiner is the microstructure characteristics of TiC and TiAl_3_ in the alloy [19]. Liu Xiangfa et al. [9] showed that the size, morphology, and distribution of TiC and TiAl_3_ particles are highly dependent on the synthesis reaction process, ultimately affected the refinement efficiency of the master alloys. Wang Zhenqing [20] investigated the kinetic parameters of phase evolution and reaction in the heating process of Al-Ti-C system by differential scanning calorimeter. They found that in the Al-40Ti-10C system, after Ti reacts with Al to form Al_3_Ti, the surplus Ti continues to react with Al_3_Ti to form a Ti-Al compound, and then it reacts with C to form TiC at about 1100 °C. Wang Liandeng et al. investigated the effect of Ce_2_O_3_ on the thermodynamics of Al-Ti-C-RE alloy which was prepared by fluoride salt method [21,22], and found that Ce_2_O_3_ can not only reduce the reaction temperature of Al-Ti-C-RE alloy but also improve the wet-ability between C and Al melt, further promoting the formation of TiC particles. Moreover, the author’s previous research found that the preparation of Al-Ti-C-Ce alloy by in-situ reaction of aluminum melt with CeO_2_ as an additive cannot only reduce the production cost, but also improve the refining performance of Al-Ti-C alloy [18,23]. However, the mechanism of rare earth oxides in the preparation of Al-Ti-C-RE alloys is still unclear [24,25,26,27,28], and there is a lack of systematic and in-depth research on the thermodynamics and kinetics of synthesis reactions.

In this paper, the Al-Ti-C-Ce alloy was prepared by the aluminum melt in-situ reaction with CeO_2_ as additive. The aim of this work was to investigate the effect of CeO_2_ on the microstructure and phase transformation of Al-Ti-C alloy during the preparation processing by quenching experiment method, based on the thermodynamic calculation of Al-Ti-C-Ce system. Finally, a macroscopic kinetic model of in-situ reaction of Al-Ti-C-Ce system was proposed.

## 2. Experimental Materials and Methods

**Figure 1:**
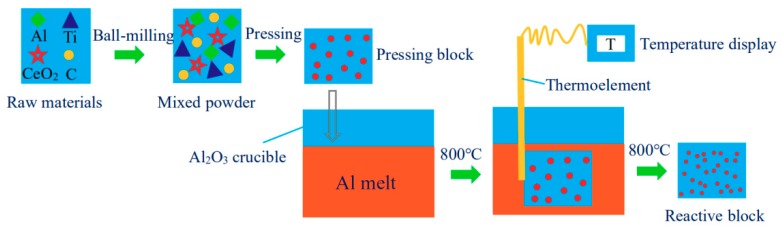
Experimental flow chart.

Al-Ti-C-Ce alloys were prepared by the aluminum melt in-situ reaction with CeO_2_ as additive, pure Al powder, pure Ti powder, and graphite powder as main raw materials. The basic parameters of the raw materials used in the experiment are shown in Table 1, and the experimental flow is shown in Figure 1. Firstly, different contents of CeO_2_, pure Al powder, pure Ti powder, and graphite powder were mixed evenly in the Pulaerisette-5 high-speed planetary with ball to material ratio = 3:1, The rotation speed and the total milling time were 350 r/min and 3 h, respectively. Then the mixed powder was pressed into a cylindrical preform of φ25 × 50 mm^2^ on an AG-10TA universal test stretching machine (Shimadzu Corporation, Kyoto, Japan), and next the preform was preheated to 200 °C in a vacuum drying oven. Simultaneously, a certain amount of commercial pure Al ingots were melted in alumina crucible by using a SG-7.5–10 type crucible furnace (Zhonghuan experimental furnace corporation, Tianjin, China). The temperature was raised to 800 °C, and then the pressing block was added into the melt. Under the heat of the high temperature Al melt, the pressing block quickly completed the reaction. The composition, the preparation temperature and numbering of each pressing block are shown in Table 2.

In order to obtain typical quenching samples at different stages of the reaction, first, the pressing blocks immersed in the Al melt were quickly taken out after holding for different time (8 s, 50 s, 60 s, 80 s, 90 s), and then were quenched in a high pressure ice brine stream. After cooling, the quenching samples were cut along the axis and then coarsely ground, finely ground, mechanical polishing, finally, electrolytic polishing by a reagent (10% HClO_4_ + 90% absolute ethanol, volume fraction, voltage 20 v). The phase of quenching samples was identified by D8 Advance X-ray diffraction (XRD, LYNXEYE detector, radius of goniometer 250 mm, the size of the five samples is 1 cm^2^, the tube has an accelerating voltage of 40 kV, an emission current of 40 mA, CuKα, λ = 1.54156 Å, scanning speed of 10°/min, step size of 0.02°, angle from 2 Theta 20° to 90°). The surface morphology and composition of the quenching samples were characterized by JSM-6700F scanning electron microscope (SEM, Shimadzu Corporation, Kyoto, Japan) and an energy dispersive spectrometer (EDS, Shimadzu Corporation, Kyoto, Japan ).

## 3. Results and Discussion

### 3.1. Thermodynamic Analysis 

The Following Reactions May Occur in the Al-Ti-C-Ce System [29,30,31]. In the above reactions, [Ti], [C], and [Ce] represent Ti atoms, C atoms, and Ce atoms in the dissolved Al melt. When the aluminum melting temperature is 1073 K, reactions (4–8) may occur in terms of thermodynamic conditions. The thermit reaction (4) is a violent exothermic reaction. Assuming a complete reaction and no heat loss under adiabatic conditions, the internal temperature of the pressing block can be made greater than 1600 K at 1073 K. At this time, △G_3_^θ^ < 0, then reaction (3) can be carried out and provides a trace amount of [C] atom to the system, so that reaction (6) is likely to occur. When the temperature T > 1553 K, △G_6_^θ^ < 0, or the temperature T > 2157 K, △G_6_^θ^ > 0, so that reaction (6) can be performed as the temperature range of the system is 1553~2157 K. From the viewpoint of thermodynamics, △G_6_^θ^ < △G_7_^θ^ < △G_8_^θ^ is in this temperature range, thus reaction (6) is most likely to happen in the Al-Ti-C system. The difference to that when CeO_2_ is added to the Al-Ti-C system, reactions (9) and (10) occur between CeO_2_ and carbon. While only when the adiabatic temperature T ≥ 1930.7 K, △G_9_^θ^ is less than zero, reaction (9) may take place spontaneously. Similarly, only when the adiabatic temperature T ≥ 1431.3 K, △G_10_^θ^ ≤ 0, reaction (10) may take place spontaneously. Since the thermit reaction (4) of synthesizing Al_3_Ti can make the local temperature of the pressing block system higher than 1600 K, reaction (10) can occur under such conditions, but it does not necessarily satisfy the thermodynamic conditions in which reaction (9) occurs. Since the solubility of C in the aluminum melt is extremely low [32,33], the tendency of reaction (6) to occur is low. Among the reaction temperature range, reaction (11) can be carried out spontaneously, and △G_11_^θ^ << △G_7_^θ^, therefore, reaction (11) is easier to synthesize TiC particles than reaction (7). This means that TiC particles can be synthesized by reaction (11) at a lower temperature. The follow reactions may occurs in the Al-Ti-C-Ce system [7,19,20,26,27,28]:

Al(s) → Al(1)△G_1_^θ^ = 10,711 − 11.48 T J/mol(1)Ti(s) → [Ti]△G_2_^θ^ = −97,166 − 13.624 T J/mol(2)C(s) → [C]△G_3_^θ^ = 71,431 − 45.970 T J/mol(3)3Al(l) + Ti(s) → Al_3_Ti(s)△G_4_^θ^ = −152,976 + 32.57 T J/mol(4)Al_3_Ti(s) → [Ti] + 3Al(l)△G_5_^θ^ = −60,897 − 26.739 T J/mol(5)[Ti] + [C] → TiC(s)△G_6_^θ^ = −162,503 + 75.314 T J/mol(6)[Ti] + C(s) → TiC(s)△G_7_^θ^ = −91,072 + 29.344 T J/mol(7)Al_3_Ti(s) + C(s) → TiC(s) + 3Al(1)△G_8_^θ^ = −41,840 − 8.898 T J/mol(8)CeO_2_(s) + 4C(s) → CeC_2_(s) + 2CO(g)△G_9_^θ^ = 771,781 − 399.74 T J/mol(9)CeO_2_(s) + 6C(s) + 2O_2_(g) → CeC_2_(s) + 4CO(g)△G_10_^θ^ = 550,699 − 384.762 T J/mol(10)CeC_2_(s) + 2[Ti] → 2TiC(s) + [Ce]△G_11_^θ^ = −527,796 + 275.905 T J/mol(11)[Ce] + 2Al_3_Ti(s) + 14Al(l) → Ti_2_Al_20_Ce(s)
(12)

### 3.2. Phase Transformation and Microstructure Transformation of Al-Ti-C-Ce System

**Figure 2:**
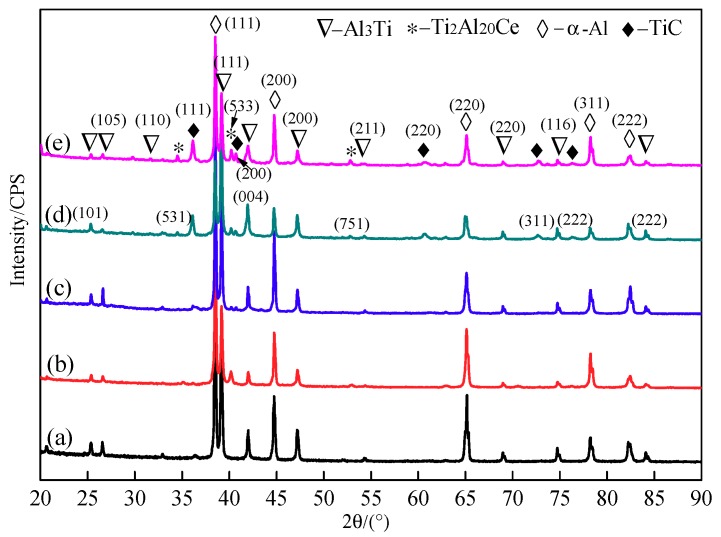
X-ray diffraction (XRD) patterns of pressing blocks complete reaction of: (**a**) 1#; (**b**) 2#; (**c**) 3#; (**d**) 4#; (**e**) 5#.

The XRD patterns of complete reaction of pressing blocks with different contents of CeO_2_ addition are shown in Figure 2. It can be seen from Figure 2a that the main phases of the 1# pressing block without CeO_2_ are α-Al, Al_3_Ti and TiC. Unlike the 1# pressing block, when CeO_2_ is added to the Al-Ti-C system, the system contains not only α-Al, Al_3_Ti and TiC, but also a rare earth phase Ti_2_Al_20_Ce. Comparing Figure 2a,b, it can be seen that when 0.5 wt.% content of CeO_2_ is added, a strong TiC diffraction peak appears at a position where diffraction angle 2θ is 41.710°, indicates that CeO_2_ is favorable for the formation of TiC particles in the Al-Ti-C system. It can be seen from Figure 2c–e that the peak intensity of TiC phase and rare earth phase Ti_2_Al_20_Ce increases gradually, while the peak intensity of Al_3_Ti phase gradually decreases with increasing CeO_2_ content.

**Figure 3:**
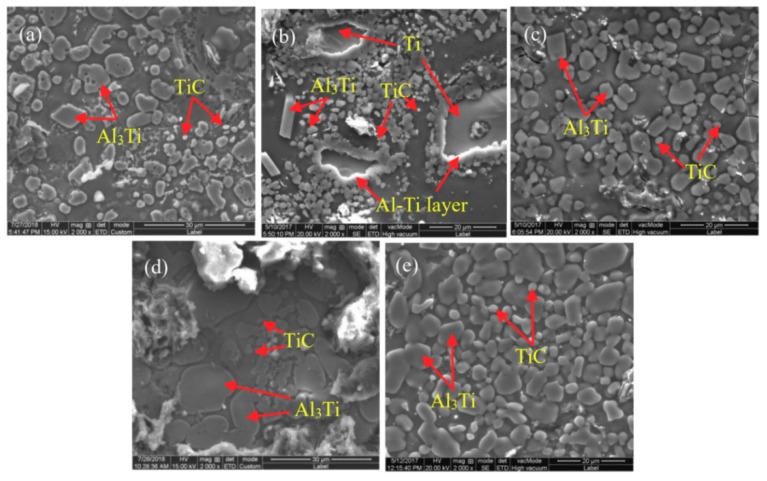
Scanning electron microscope (SEM) images of pressing blocks complete reaction of: (**a**) 1#; (**b**) 2#; (**c**) 3#; (**d**) 4#; (**e**) 5#.

The SEM images of complete reaction of pressing blocks with different contents of CeO_2_ addition are shown in Figure 3. According to the XRD patterns and the EDS spectrum analysis, we judge that the block-like particles are Al_3_Ti, which has a size of about 8 μm, and the small particles are TiC, which has a particles size of about 2 μm in Figure 3a. As shown in Figure 3b, it can be seen that when the CeO_2_ content is 0.5%, the shape of Al_3_Ti particles in the 2# pressing block is block-like and rod-like. As compared to the 1# sample, the TiC particles increase but the distribution is inhomogenous. As shown in Figure 3b–e, the number of TiC particles gradually increase with CeO_2_ contents. when the CeO_2_ content reaches 4%, the shape of Al_3_Ti and TiC particles in the 5# pressing block are relatively regular and the number are relatively large, the TiC particles are dispersal distributed and the size is homogeneous.

**Figure 4:**
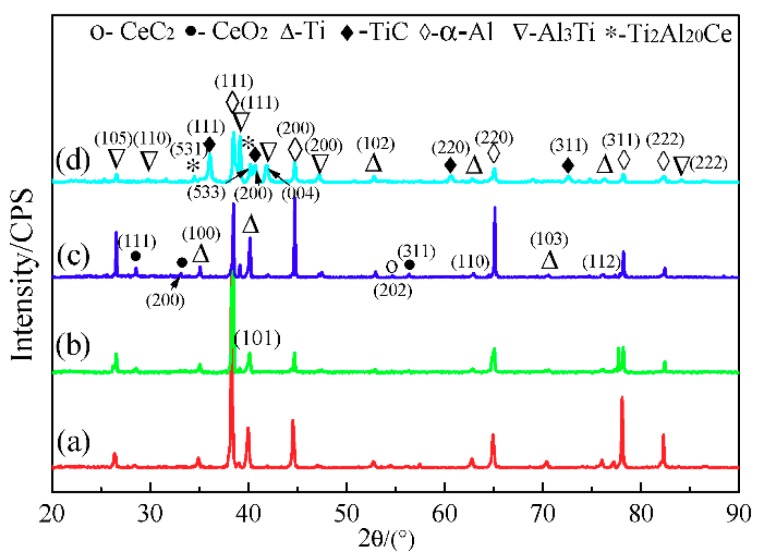
XRD patterns of pressing blocks at 60 s of: (**a**) 2#; (**b**) 3#; (**c**) 4#, and (**d**) 5#.

The XRD patterns of pressing blocks with different contents of CeO_2_ at 60 s are shown in Figure 4. It can be seen from Figure 4a–c that there are diffraction peaks of Ti when the amount of CeO_2_ added is low, indicating that a small amount of Ti(s) in the system which is not involved in the reaction. As CeO_2_ contents increases, the diffraction peaks intensity of Al_3_Ti and TiC phases gradually increase. When the CeO_2_ content reaches 4%, the rare earth phase Ti_2_Al_20_Ce forms in the 5# pressing block. At this time, the Al_3_Ti phase diffraction peak intensity of 5# sample is weaker than that of the 4# sample, but the TiC phase diffraction peak intensity is obviously enhanced. This can be contributed to the fact that reaction (12) forms the rare earth phase Ti_2_Al_20_Ce, which is based on the Al_3_Ti reacted with [Ce] produced by reaction (11), thus consuming the amount of Al_3_Ti.

**Figure 5:**
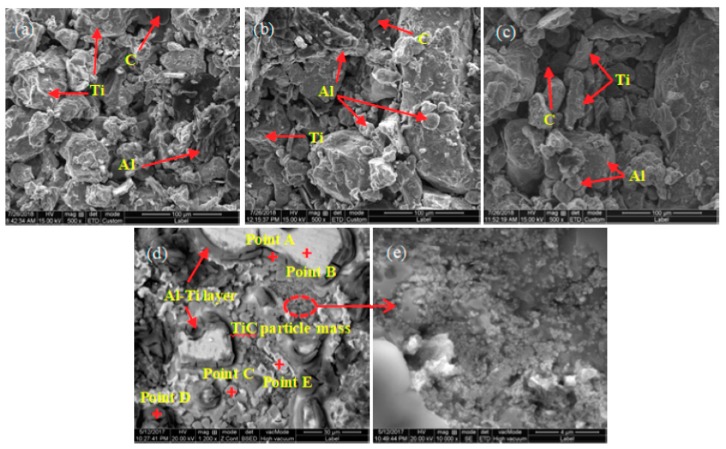
SEM images of pressing blocks at 60 s of: (**a**) 2#; (**b**) 3#; (**c**) 4#; (**d**) 5#; (**e**) TiC particle mass in Figure 5d.

The SEM images of pressing blocks with different CeO_2_ contents at 60 s are shown in Figure 5. The EDS spectrums of each point in Figure 5d are shown in Table 3. It can be seen from Figure 5a to (e) that under the same reaction time (60 s), the internal reaction degree of the pressing block increase gradually with CeO_2_ contents. When the CeO_2_ content is 4%, the 5# pressing block is near the state of complete reaction. Combined with XRD patterns and EDS analysis, it can be seen that the large bright white particles in Figure 5d are Ti particles, and an Al-Ti layer is formed around the Ti particles. Block-like Al_3_Ti particles with a size of ~5 μm are distributed around the Al-Ti layer. A small amount of bright white block-like Ti_2_Al_20_Ce exists in the vicinity of the Al_3_Ti particles. A large amount of TiC clusters is distributed between the Al_3_Ti and Ti_2_Al_20_Ce particles.

**Figure 6:**
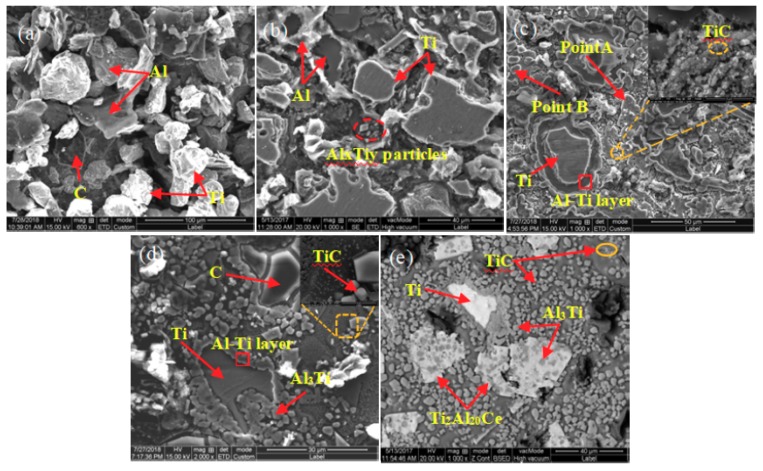
SEM images of 5# pressing block reaction at different time: (**a**) 8 s; (**b**) 50 s; (**c**) 60 s; (**d**) 80 s, and (**e**) 90 s.

**Figure 7:**
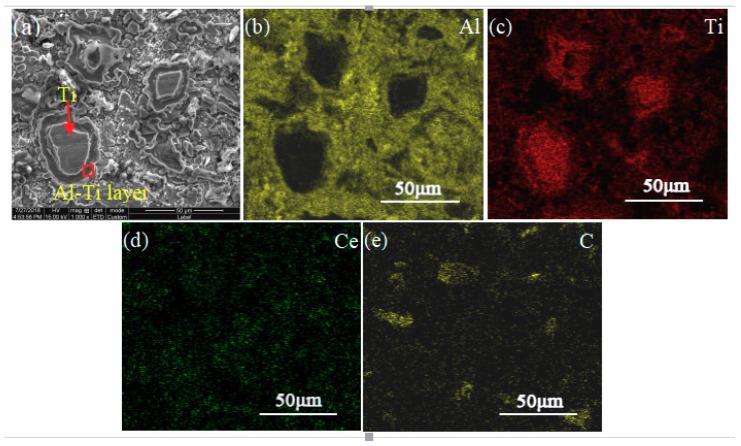
Map scan patterns of Figure 6c: (**a**) Is the SEM image of Figure 6c, (**b**–**e**) are map scan patterns of Al, Ti, Ce, and C, respectively.

**Figure 8:**
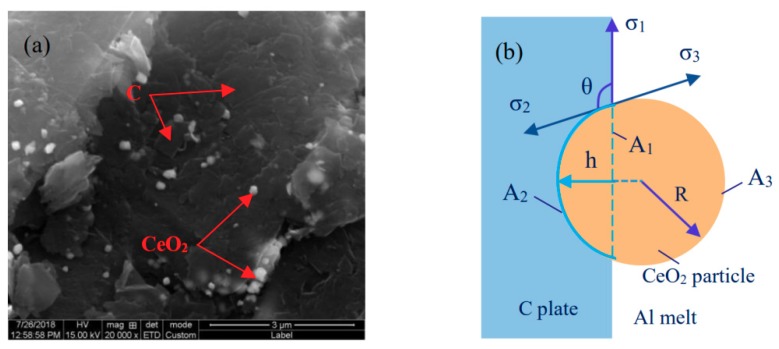
SEM image and model diagram of CeO_2_ particles adsorbed on the surface of C sheet in aluminum melt: (**a**) SEM spectrum of 5# pressing block at reaction time of 50 s; (**b**) schematic diagram of the model.

In order to further research the effect of CeO_2_ on the phase transformation and microstructure transformation of Al-Ti-C system, the 5# pressing block was selected and the microstructure under different reaction time was studied. The SEM images are shown in Figure 6. Table 4 shows EDS composition analysis of point A and point B in Figure 6c. Figure 7 shows the map scan patterns of Figure 6c. As can be seen from Figure 6a, at the initial stage of reaction (8 s), the system remains substantially in the original particle state. As shown in Figure 6b, when the reaction time reaches 50 s, Ti particles are surrounded by the melting Al, and a small amount of AlxTiy particles [3,9] is presented in the system, indicating that a small amount of reaction occurs in the system at this time. Combined with the results of the point scan spectrums analysis of Figure 6c as shown in Table 4, the results of Ti:Al:Ce is close to 2:20:1, and Al:Ti is close to 3:1, hence it can be judged that large block-like particles with size of about 30 μm are rare earth phase Ti_2_Al_20_Ce, and little block-like particles with size of about 5 μm are Al_3_Ti. As can be seen from Figure 8, Ti particles are wrapped in Al solution, and some Ti and Ce elements are around Ti particles in Al solution. In addition, Ce elements are also enriched in C rich area, and Ti elements also around C particles. Thus, when the reaction time reaches 60 s, the cladding structure of Al/Al-Ti/Al_3_Ti/Ti_2_Al_20_Ce is formed in the system [26,28], and a large amount of TiC particles is formed around the cladding structure. It can be seen from Figure 6c–e that as the reaction time prolongs, the Al_3_Ti particles form free particles from the cladding structure, the TiC particles gradually grow, and the cladding structure gradually disappears. In Figure 6e, the small block-like particles with an average size of about 6 μm are Al_3_Ti, the small particles with a size of about 1 μm are TiC, and the bright white block-like particles with a size of about 30 μm are rare earth phase Ti_2_Al_20_Ce. As can be seen from Figure 6e, a large amount of Al_3_Ti particles is surrounded by the rare earth phase Ti_2_Al_20_Ce. Thus the rare earth phase Ti_2_Al_20_Ce is formed by reaction (12), that is, the Al_3_Ti particles are used as core nucleation, and reacts with [Ce] which is enriched around them [26,34].

Further analysis of the quenched sample of the 5# pressing block at 50 s shows that many CeO_2_ particles are adsorbed on the surface of the C particles as shown in Figure 8a. According to Figure 8a, a schematic diagram of the model for adsorbing CeO_2_ particles on the surface of C sheets in Al melt is established in Figure 8b (RCeO_2_ << RC in the experiment, so the contact surface between CeO_2_ particles and C sheets is assumed to be in plane). As show in Figure 8b [35], σ_1_, σ_2_, and σ_3_ are the interfacial energy of the Al/C interface, the C/CeO_2_ interface, and the Al/CeO_2_ interface, respectively, A_1_, A_2_, and A_3_ are their contact areas, respectively, and R is the radius of the CeO_2_ particles. h is the adsorption depth of CeO_2_ particles, and θ is the contact angle of CeO_2_ particles at the C/CeO_2_ interface. It can be seen from Figure 8b that when CeO_2_ particles are adsorbed on the surface of C particles, the total interfacial energy ΔGs is composed of three parts: one is the reduction of Al/C interfacial energy, G_1_ = −σ_1_A_1_; the second is C/CeO_2_ interface energy increase, G_2_ = σ_2_A_2_; the third is the increase of Al/CeO_2_ interface energy, G_3_ = σ_3_A_3_.

The interface energy can be numerically represented by the value of the surface tension. When this state is stable in the melt, the three surface tensions reach equilibrium at the intersection [35,36], namely:

σ_2_ = σ_3_ + σ_1_cosθ; cosθ = (σ_2_ − σ_3_)/σ_1_

According to the geometric relationship, the values of h, A_1_, A_2_, and A_3_ can be solved as follows:

h = R(1 + cosθ); A_1_ = πR^2^(1 − cos^2^θ);

A_2_ = 2πR^2^(1 + cosθ); A_3_ = 2πR^2^(1 − cosθ)

Then the total interface energy changes are:

△Gs = G_2_ + G_3_ − G_1_ = 2πσ_2_R^2^(1 + cosθ) + 2πσ_3_R^2^(1 − cosθ) + πσ_1_R^2^(1 − cos^2^θ)

= πR^2^[2(σ_2_ + σ_3_) + 2(σ_2_ − σ_3_)cosθ + σ_1_(1 − cos^2^θ)]

When the CeO_2_ particles are not adsorbed into the surface of the C particles, and the particles are close to the C or Al melt, the total interfacial energy G’ = 4πσ_2_R^2^ or 4πσ_3_R^2^, however, when the CeO_2_ particles are adsorbed on the surface of the C particles, the binding energy is:

ΔG = G′ − ΔGs = −πR^2^σ_1_(cosθ ∓ 1)^2^

The above formula shows that the CeO_2_ particles are more preferentially adsorbed on the surface of the C particles in the aluminum melt. Due to the strong surface activity and “catalytic effect” of CeO_2_ [21,37,38], and a large number of bubbles appear in the aluminum melt during the experiment, combined with the thermodynamic calculations in Section 3.1, it can be judged that reaction (10) occurs between C and CeO_2_ adsorbed on the surface of the C particles, so that CO gas was produced. The occurrence of reaction (10) creates conditions for reaction (11) of the TiC particles.

### 3.3. Kinetic Analysis

**Figure 9:**
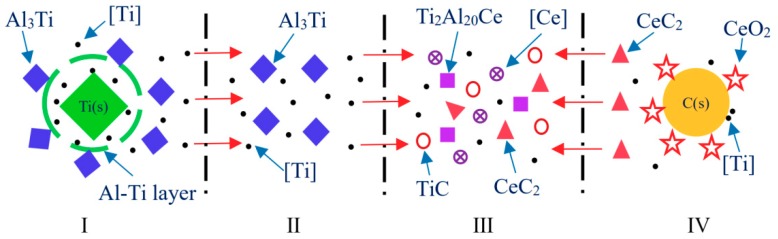
Microscopic kinetics mechanism model of TiC synthesis in Al-Ti-C-Ce system. I: 3Al(l) + Ti(s) → Al_3_Ti(s); II: Al_3_Ti(s) → [Ti] + 3Al(l); III: CeC_2_(s) + 2[Ti] → 2TiC(s) + [Ce]; IV: CeO_2_(s) + 6C(s) + 2O_2_(g) → CeC_2_(s) + 4CO(g).

The microscopic kinetics model of TiC synthesis in Al-Ti-C-Ce system was established by thermodynamic analysis of Al-Ti-C-Ce system and microstructural analysis of typical quenched samples at different stages of the reaction, as shown in Figure 9. The synthesis of TiC can be microscopically divided into four microdomains: The first is the formation of Al_3_Ti particles. When the pressing block is placed in the aluminum melt, the aluminum melt penetrates into the pressing block so that the internal temperature of the pressing block rises and the aluminum powder melts and wraps around the surface of the Ti particles. An Al-Ti layer is formed around the Ti particles by solid-liquid diffusion, and a reaction (4) 3Al(l) + Ti(s) → Al_3_Ti(s) occurs. The second is the dissolution of Al_3_Ti particles. Since reaction (4) is a severe exothermic reaction, the Al_3_Ti particles are separated from the Al-Ti layer as the reaction progresses. The Al_3_Ti particles undergo a dissolution reaction (5) Al_3_Ti(s) → [Ti] + 3Al(l) under high temperature to produce [Ti]. It migrates to the III microdomain and provides [Ti] for the formation of TiC particles. The fourth is mainly a carbothermal reaction of CeO_2_ and C. After ball milling, CeO_2_ particles are adsorbed onto the surface of C particles. When a certain temperature is reached, CeO_2_ reacts with C, and CeC_2_ produced by reaction (10) CeO_2_(s) + 6C(s) + 2O_2_(g) → CeC_2_(s) + 4CO(g) migrates to the third microdomain. The third microdomain is mainly the formation of TiC particles. As the reaction progresses, [Ti] produced by the dissolution of the Al_3_Ti particles reacts with CeC_2_ generated in the IV microdomain, and reaction (11) CeC_2_(s) + 2[Ti] → 2TiC(s) + [Ce] generates TiC particles.

**Figure 10:**
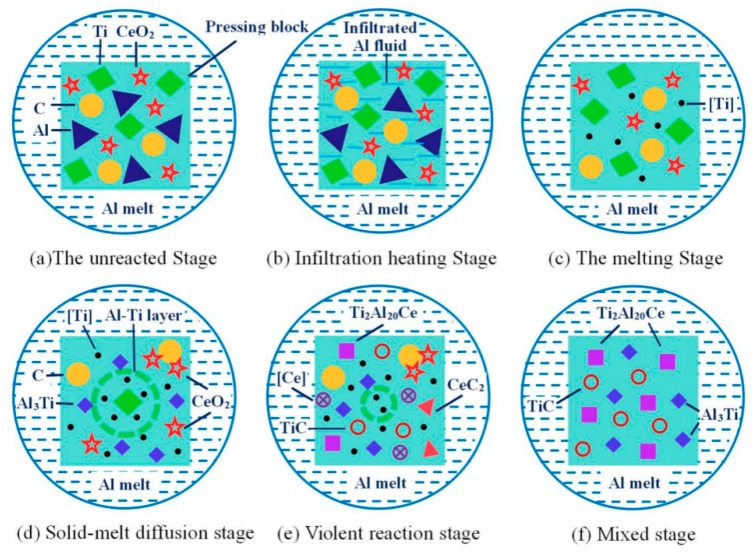
The dynamic model of the Al-Ti-C-Ce system.

Through the above analysis, a macroscopic kinetic model of the Al-Ti-C-Ce system reaction was established, as shown in Figure 10. The reaction process of the whole system is mainly divided into four stages: The infiltration heating stage, the melting stage, the solid-liquid diffusion stage, and the complete reaction stage.

Infiltration heating stage: As shown in Figure 10a,b, the pressing block is added to the aluminum melt. First, the aluminum melt penetrates into the interior of the pressing block, so that the internal temperature of the system rises. Melting stage: As shown in Figure 10b,c, when the internal temperature of the pressing block reaches the melting point of aluminum, the aluminum melts and spreads rapidly on the surface of the Ti particles and the C particles. At the same time, the Ti particles also dissolve and diffuse into the aluminum melt. Graphite still exists in solid form. Reactions (1) and (2) occur mainly at this stage.

Solid-liquid diffusion stage: As shown in Figure 10c,d, as the reaction progresses, Al and Ti wrapped on the surface of the Ti particles react by solid-liquid diffusion to form Al_3_Ti. The Al_3_Ti particles are separated from the Al-Ti layer under high temperature, and a dissolution reaction (5) occurs to form [Ti].

Complete reaction stage: As shown in Figure 10d,e, CeO_2_ reacts with C under O_2_ to form CeC_2_ and CO, then CeC_2_ reacts with [Ti] produces TiC. The [Ce] produced by reaction (11) is then reacted with Al_3_Ti to form Ti_2_Al_20_Ce.

## 4. Conclusions

(1) The addition of CeO_2_ is beneficial to promote the formation of TiC particles in the Al-Ti-C system. With increasing CeO_2_ content, the number of TiC particles increases, and the rare earth phase Ti_2_Al_20_Ce is formed.

(2) CeO_2_ has little effect on the synthesis of Al_3_Ti particles in the Al-Ti-C system. Al_3_Ti is mainly formed by solid-liquid diffusion at the interface between molten Al and Ti particles.

(3) CeO_2_ has an important influence on the synthesis of TiC particles. In the Al-Ti-C system, TiC is mainly formed by the reaction of dissolved [Ti] and C(s) in the system. In the Al-Ti-C-Ce system, CeO_2_ first reacts with C and O_2_ to form CeC_2_, and then CeC_2_ reacts with dissolved [Ti] to form TiC.

## Figures and Tables

**Table 1:** Characteristics of materials.

Materials	Grain Size/μm	Purity/%
Al powder	78–104	99.0
Ti powder	44–68	99.0
C powder	12–22	99.0
CeO_2_ powder	2–4	99.9
Commercially pure Al	-	99.7

**Table 2:** The preparation parameters of different prefabricated blocks.

Sample No.	Composition of Prefabricated Blocks	Preparation Temperature/K
1#	Al-Ti-C	1073
2#	Al-Ti-C + 0.5 wt.%CeO_2_	1073
3#	Al-Ti-C + 1.0 wt.%CeO_2_	1073
4#	Al-Ti-C + 2.0 wt.%CeO_2_	1073
5#	Al-Ti-C + 4.0 wt.%CeO_2_	1073

Note: The total mass of each sample is 50 g, Al:Ti:C = 5:2:1 is the molar ratio.

**Table 3:** Energy dispersive spectrometer (EDS) composition analysis of point A, point B, point C, point D, and point E in Figure 5d.

Point No.	Atomic (Al)/%	Atomic (Ti)/%	Atomic (C)/%	Atomic (Ce)/%
A	49.7	50.3	-	-
B	-	100	-	-
C	76.2	23.8	-	-
D	95.1	4.9	-	-
E	76.2	11.1	8.0	4.7

**Table 4:** EDS composition analysis of point A and point B in Figure 7c.

Point No.	Atomic (Al)/%	Atomic (Ti)/%	Atomic (C)/%	Atomic (Ce)/%
A	86.6	9.2	-	4.3
B	65.5	21.5	13.0	-
